# On Watering Streets

**Published:** 1850-10

**Authors:** Theodore S. Bell

**Affiliations:** Louisville, Ky.


Art. II.—*On Watering Streets.* By Theodore S. Bell, M. D., of Louisville, Ky.

I cannot believe that an evil temper, and abusive language are capable of advancing the interests of medical science, nor that they are conducive to a philosophical investigation, and in examining a paper written by Dr. S. Jackson, for the September number of the *Medical Examiner*, and entitled: "Friendly hints to a Reviewer," I shall treat it with as much deference and respect as though it were entitled to be approached with those feelings. No other spirit than that of an inquirer after truth, or of an advocate for it, should ever animate a medical writer, and truth needs none of the extraneous aids of anger, nor abusive language.

The subject of hygiene is one that deserves the best efforts of medical minds. It is a basis upon which medical science may build a superstructure that will bid defiance to all the angry surges that may be sent against it—the foundation is a rock, and cannot be moved. The cultivation of public hygiene, too, seems to mark the boundary line between the empire of true medical science, and the narrow domains of empiricism. We know of no system of quackery, from the days of Perkins's tractoration, down to the most recent phase of empiricism that makes any attempt to guard the people from the causes of disease. The devotees of the various systems of quackery, in the midst of boundless pretension to the cure of all diseases, seem to prefer the prevalence of sickness, and act as though they considered cure better than prevention.

In a proper spirit of investigation I undertook, in the July number of this Journal, to examine a paper, "On Wetting the Streets," read by Dr. S. Jackson, before the Philadelphia County Medical Society. I weighed the statements of that paper carefully, and found them wanting in some essential elements of truth. And Dr. Jackson's article in response has been fully examined, without finding that its author has either fortified any one of his positions, or advanced the interests of a principle for which he contends. No proof has been furnished which shows that sprinkling the streets of a city, with pure water, has any deleterious effect. The "story" of Capt. Murray's ship, the capital on which extensive operations are attempted, does not prove that the drying operations referred to, had any special agency in preserving the health of the crew of his vessel. Various other agencies are detailed by Capt. Murray, and they are entitled to a more conspicuous place in Dr. Jackson's reasonings, than his *asserted*, but unproved dryness of the atmosphere of the ship. As a proof of this fact, we know that Capt. Ross accomplished the same results that Capt. Murray claims, in the same regions that Capt. M.'s ship occupied, without the use of brodiestoves, or any substitute for them. If the same result is attained without the use of a special agent, that was gained in the company of that agent, we are safe in saying it had nothing to do with the result.

In proof of the fact that a mere humid condition of the atmosphere is not essentially detrimental, the observations of medical men, on the effects of fogs were referred to. Surely a foggy atmosphere is humid enough. But Dr. Jackson takes fire at this, and in his heat strikes off the following: "But why name fogs, *a word that is not to be found in my whole paper.*" A little coolness and calmness might have saved Dr. Jackson from this statement. Let him compare the assertion italicised in the above quotation, with the following extract from page 327 of his original essay: "In every campaign, he says," [Dr. Pringle,] "no epidemic ever ensued on the greatest heats till the perspiration was stopped by wet clothes, wet beds, dews, or *fogs.*" That looks something like finding the word fogs in Dr. Jackson's paper.

There is too much of this kind of assertion in Dr. Jackson's "Hints to a Reviewer." Take another instance. Of the statement made in my former paper—"the humid state of Torquay is notorious," Dr. Jackson says: "Sir James Clarke, the highest authority says otherwise," and Dr. Clarke, quoted by Dr. Jackson, proves just what I said. Read him—"hence while Torquay possesses all the advantages of the climate of this coast, its chief disadvantage, *humidity*, is felt in a less degree than elsewhere."

Dr. James Johnson, a name that will be held in reverence, as long as medicine is cultivated as a science, thus writes on dry and humid atmospheres: "The very circumstance, in short, which forms the charm, the attraction, the theme of praise in the Italian climate, is that which renders it dangerous, because deceitful—namely, the *long intervals of fine weather between vicissitudes of great magnitude.* This is the bane of Italy, whose brilliant suns, and balmy zephyrs flatter only to betray."

Again: "Italy boasts much of the dryness of her climate. In some places, as at Pisa, there falls as much rain as in Cornwall. In Rome, about one-third less of rain falls than at Penzance, and the number of rainy days is one-third less—being about 117 in the year. This is a poor counterbalance for the *steam* of the sirocco, and the oppressive stillness of the Roman air. *The fogs of England* and its cloudy skies furnish constant themes of querulous complaint; but they would be rich treats in Italy, as defences against the torrents of liquid fire that pour down on her vales from a nearly vertical sun."—*Change of Air*, p. 262.

If it is objected that the sirocco of Italy is a hot moist air, let it be remembered that it is not as terrific in its effects as the hot, dry simoom of the Lybian desert.

In answer to the statement that invalids in the West India islands, were advised by Dr. James Clark to spend their time on the water, Dr. Jackson says: "As to the people seeking their safety on the water, this is merely the less of two evils, for surely no one is crazy enough to pretend that a ship in the torrid zone is a healthy situation." Now Dr. James Clarke, announced by Dr. Jackson, as "the highest authority" advises that invalids may be sent to the West Indies, provided they spend their time on the water. And if any one is unfortunately the victim of lunacy for saying that a ship in the torrid zone is a healthy situation, what becomes of the "story" of Capt. Murray's ship, which plays such a conspicuous part in Dr. Jackson's two papers? When Capt. Murray's ship appears to sustain Dr. Jackson's hypothesis, the high health of the officers and crew in a torrid zone, is triumphantly appealed to, but when I give Sir James Clake's statement, that invalids may reap advantage by visiting the West India islands, provided they live on the water, Dr. Jackson declares that no body but a crazy man would say that a ship in the torrid zone is "a healthy situation." Alas! the legs of the lame are not equal.

Dr. Jackson quotes Dr. Yandell's very correct remarks about the condition of the streets of Louisville, as an answer to my statement that the streets most constantly wet are the healthiest streets. This is rather straining the matter. There is no contradiction between the statements made by Dr. Yandell, and those I made. He refers to the rapid drying after rains. I wrote of streets that are kept wet by water carts. My facts are fatal to Dr. Jackson's hypothesis, and I repeat them. Large portions of Main and Market streets, and a number of the cross streets that intersect them are kept almost constantly wet, through the summer, and make quite as respectable an approach towards the manufacture of an Italian sirocco, as Philadelphia does. These streets are much more healthy than a large, dry area in the suburbs is at this time, and surely more of Dr. Jackson's "steam" is made on the immense wet surface I have described as the healthy district, than can be made by one or two small marshes, which are to be found in the large dry area. If Dr. Jackson can make his hypothesis tally with these facts, it behooves him to do so. If he cannot he must acknowledge his error, and yield the question. He must meet the facts with truth, and not attempt to cover his retreat, by unwarrantable interpretations, of what he calls "cant phrases" among Western men, and by perverting language to suit his own views. When I said "a considerable part of the city retains its health," I meant just what a correct Philadelphia medical writer would mean by the same language—I said, and meant that the considerable portion of the city referred to, enjoyed good health—there was not sickness of various kinds in every part of the city, as Dr. Jackson's imagination induces him to represent the fact.

I have thus answered the leading points of Dr. Jackson's second paper on wetting streets. There are various things in that paper, which have no kind of bearing on the hygienic question before us, and I shall let them pass to that inevitable bourne for which they are fitted. There are wild and reckless assertions too, such as his denial of his admission that streets may be safely sprinkled with pure water, upon which it is unnecessary to waste time. But I regret that in Dr. Jackson's anxiety to be fierce, he fires a broadside at the city of Louisville, and endeavors to show that the people are graceless outlaws. He says:

"Have not the people of Louisville built a huge bridge which the law will soon compel them to unbuild? This however is not so much a murmuring as an open rebellion, for the New Testament enjoins all men to obey the laws." Now Dr. Jackson is respectfully informed that Louisville has not built the huge bridge of which he speaks. That bridge is at Wheeling, Virginia, which is several miles from Louisville. But what this had to do with the hygienic question, I am at a loss to know. It is, however, in perfect keeping with that medley of Greek, Latin, French, and—I am at a loss to name the other language, that flourishes over nearly sixteen pages of the Medical Examiner, under the name of Dr. S. Jackson.

